# The neural correlates of the awe experience: Reduced default mode network activity during feelings of awe

**DOI:** 10.1002/hbm.24616

**Published:** 2019-05-07

**Authors:** Michiel van Elk, M. Andrea Arciniegas Gomez, Wietske van der Zwaag, Hein T. van Schie, Disa Sauter

**Affiliations:** ^1^ Department of Psychology University of Amsterdam Amsterdam the Netherlands; ^2^ Amsterdam Brain and Cognition Center University of Amsterdam Amsterdam the Netherlands; ^3^ Spinoza Center for Neuroimaging Royal Netherlands Academy of Arts and Sciences Amsterdam the Netherlands; ^4^ Behavioral Science Institute Radboud University Nijmegen Nijmegen the Netherlands

**Keywords:** absorption, awe, default mode network, fMRI, frontoparietal network

## Abstract

In the present fMRI study, we aimed to obtain insight into the key brain networks involved in the experience of awe—a complex emotion that is typically elicited by perceptually vast stimuli. Participants were presented with awe‐eliciting, positive and neutral videos, while they were instructed to get fully absorbed in the scenery or to count the number of perspective changes. By using a whole‐brain analysis we found that several brain regions that are considered part of the default mode network (DMN), including the frontal pole, the angular gyrus, and the posterior cingulate cortex, were more strongly activated in the absorption condition. But this was less the case when participants were watching awe videos. We suggest that while watching awe videos, participants were deeply immersed in the videos and that levels of self‐reflective thought were as much reduced during the awe videos, as during the perspective counting condition. In contrast, key regions of the fronto‐parietal network (FPN), including the supramarginal gyrus, the medial frontal gyrus, and the insula, were most strongly activated in the analytical condition when participants were watching awe compared to positive and neutral videos. This finding underlines the captivating, immersive, and attention‐grabbing nature of awe stimuli that is considered to be responsible for reductions in self‐reflective thought. Together these findings suggest that a key feature of the experience of awe is a reduced engagement in self‐referential processing, in line with the subjective self‐report measures (i.e., participants perceived their self to be smaller).

## 1 INTRODUCTION

As many renowned scientists, ranging from William James to Albert Einstein and Richard Dawkins, have noted, awe is an overwhelming emotion that is at the basis of religion, great scientific achievements, and magnificent works of art (Dworkin, 2013). Awe is typically elicited when we are confronted with perceptually vast natural objects, such as mountains, vistas and oceans (Fuller, 2006; Shiota, Keltner, & Mossman, 2007), but can also be elicited by powerful and engaging music (Maruskin, Thrash, & Elliot, 2012; Salimpoor, Benovoy, Longo, Cooperstock, & Zatorre, 2009), intense meditative practice (Reinerman‐Jones, Sollins, Gallagher, & Janz, 2013), impressive man‐made objects such as skyscrapers (van Elk, Karinen, Specker, Stamkou, & Baas, 2016) or videos in which the timing of physical and natural phenomena is either slowed down (e.g., seeing a droplet fall down; cf., Piff, Dietze, Feinberg, Stancato, & Keltner, 2015) or speeded up (e.g., seeing flowers rapidly grow).

Several studies have focused on the *causes* of awe, such as the elicitors and personality factors predisposing people to experience awe (Shiota et al., 2007; Tam, 2013). This work has established that people differ in their propensity for having awe‐like experiences (Shiota et al., 2007) and that openness to experience and the personality trait of “absorption” are strong predictors of the intensity of awe experiences (Silvia, Fayn, Nusbaum, & Beaty, 2015; van Elk et al., 2016). Other studies have investigated the behavioral *consequences* of awe, indicating for instance that awe can increase prosocial behavior (Piff et al., 2015; Schnall, Roper, & Fessler, 2010; Weinstein, Przybylski, & Ryan, 2009; Zhang, Piff, Iyer, Koleva, & Keltner, 2014), environmental awareness (Kamitsis & Francis, 2013; Tam, 2013), and well‐being (Howell, Dopko, Passmore, & Buro, 2011; Rudd, Vohs, & Aaker, 2012; Zhang, Howell, & Iyer, 2014).

Awe and perceived vastness may also cause a reduced focus on and awareness of one's self (Piff et al., 2015; van Elk et al., 2016; Zhang, Piff, et al., 2014). Several studies have reported that perceptually vast and awe‐eliciting stimuli can induce the feeling of a “small self”, characterized by a reduced focus on the self and its related concerns (Bockelman, Reinerman‐Jones, & Gallagher, 2013; Campos, Shiota, Keltner, Gonzaga, & Goetz, 2013; Piff et al., 2015; Reinerman‐Jones et al., 2013; van Elk et al., 2016). The notion that awe induces changes in the perception of the self is also supported by studies on the effects of natural environments (for review, see: Bratman, Hamilton, & Daily, 2012). Nature is a strong elicitor of awe (Shiota et al., 2007) and it has been reported that esthetic experiences of nature result in a diminished focus on the self and in stronger prosocial behavioral tendencies. For instance, the immersion in natural landscapes has been shown to result in enhanced generosity, helping behavior (Zhang, Howell, & Iyer, 2014; Zhang, Piff, et al., 2014) and moral care (Weinstein et al., 2009). In all these cases, the psychological mechanism underlying the effects of awe on prosocial behavior is likely a process of “unselfing” (Murdoch, 1967). This process allows one to go beyond self‐interest by shifting the focus away from oneself and toward others and the outside world (Piff et al., 2015).

To date there is only indirect evidence for the notion that awe is associated with a reduced focus on the self, because most behavioral studies have relied entirely on self‐report measures. Typically, participants retrospectively assess their subjective feelings of awe following an experimental manipulation. However, it has been argued that such self‐report measures are prone to different biases (e.g., transient mood states; demand characteristics; respondents' use of implicit theories; cf. Podsakoff, MacKenzie, Lee, & Podsakoff, 2003). The observed relation between awe and a decreased focus on the self may also be partly related to the common method bias, because of a conceptual overlap between the items used to measure awe (e.g., “I felt a loss of sense of space and time”) and those used to measure the “small self” (e.g., “I felt the presence of something greater than myself”; Podsakoff et al., 2003).

In the present study we used functional magnetic resonance imaging (fMRI) as a way to measure brain activity *during* the experience of awe, to obtain insight into the neurocognitive mechanisms underlying the awe experience. This has the advantage of providing a measure that does not risk being influenced by memory biases, as well as more broadly avoiding the problems inherent in self‐report measures.

Based on the phenomenological reports suggesting that awe is characterized by a reduced focus on the self, we hypothesized that the feeling of awe would be accompanied by a relative decrease of activation of brain areas that are considered to be part of the so‐called Default Mode Network (DMN)—a network of brain regions that has been mainly implicated in self‐referential processing (Qin & Northoff, 2011) and mind‐wandering (Fox, Spreng, Ellamil, Andrews‐Hanna, & Christoff, 2015). Different subdivisions have been distinguished within the DMN, such as the core DMN (consisting of the anterior MPFC, the PCC and the posterior IPL), the subsystem of the DMN centered around the medial temporal lobe (including the hippocampal formation and the parahippocampal cortex) and a third subsystem (including the dorsomedial component of the PFC, the inferior frontal gyrus and the lateral temporal cortex; Buckner, Andrews‐Hanna, & Schacter, 2008). These different subcomponents and regions have also been associated with different cognitive processes. The MPFC has been specifically associated with the processing of self‐related information (Qin & Northoff, 2011) and the PCC seems primarily involved with getting caught up in one's experience (Brewer, Garrison, & Whitfield‐Gabrieli, 2013). Thus, we hypothesized that during the experience of awe participants would be captivated by their present experience, which should result in less self‐referential processing and an accompanying reduction in activity of key regions of the DMN, such as the MPFC and the PCC.

Neuroimaging studies focusing on psychedelics, flow and meditation provide indirect support for the hypothesis that the experience of awe is accompanied by a reduced activation of the DMN. For instance, several recent studies have used psychedelic drugs, such as psilocybin or LSD, to induce experiences of “ego dissolution” (i.e., the disappearance of the notion of a core‐self), showing that this experience is accompanied by a reduced activation of the DMN (Lebedev et al., 2015; Palhano‐Fontes et al., 2015) and decreased functional connectivity within the DMN (Palhano‐Fontes et al., 2015; Tagliazucchi et al., 2016). Furthermore, experimentally induced flow (i.e., a state of perceived fit between skills and task demands while engaging in a particular activity, such as gaming) is also associated with decreased activation in key regions of the DMN (Ulrich, Keller, Hoenig, Waller, & Gron, 2014). Finally, peak‐experiences of meditation that are characterized by a reduced awareness of the body, space and time are also associated with a decreased activation of the DMN (Brewer et al., 2011). It has been argued that a shared characteristic of these different types of experiences is a reduced salience of the self (Yaden, Haidt, Hood, Vago, & Newberg, 2017), suggesting a common mechanism underlying self‐transcendental experiences, including feelings of awe. In sum, based on both behavioral and neuroimaging studies we hypothesized that the experience of awe would be characterized by a reduced awareness of the self and an accompanying decreased activity of core regions of the DMN (Raichle, 2015), such as the precuneus and the medial prefrontal cortex (MPFC).

**Figure 1:**
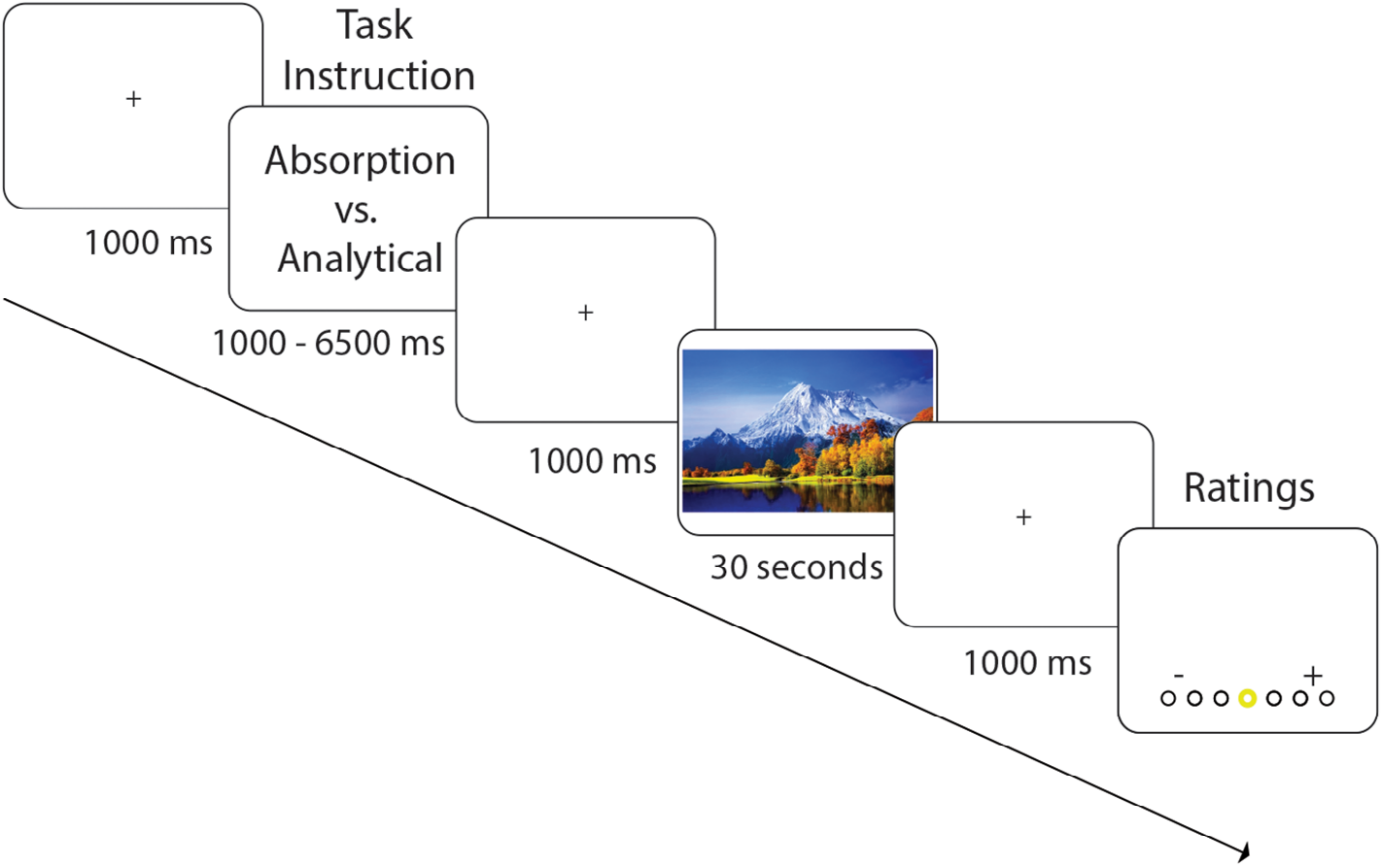
Experimental design and procedure. Participants were presented with three different types of videos (awe, positive, or neutral) in either the absorption instruction condition or the analytical instruction condition. During each trial, participants were first presented with a task instruction, followed by a 30‐s video and following the video they were required to rate the video for feelings of awe, arousal, and valence and to report the number of perspective changes (in the analytical condition) [Color figure can be viewed at http://wileyonlinelibrary.com]

To test our hypothesis, we conducted an fMRI study in which participants were presented with videos of natural phenomena that in previous studies have been used successfully to elicit feelings of awe (Piff et al., 2015; van Elk et al., 2016). As control stimuli we used *neutral videos* of landscapes and *positive videos* of funny animals. The positive control stimuli were matched to the awe stimuli (in a pretest) in terms of perceived valence and arousal, while they differed in the potential to elicit feelings of awe. To control for the potential confound that systematic differences between the content of the different types of videos would contribute to differences in brain activation between conditions, we manipulated the mindset with which participants were watching the videos. Prior to each video we instructed participants to count the number of visual perspective changes in the video (we labeled this the “analytical condition”) or to get passively immersed in the video (i.e., as if watching the video in a cinema; we labeled this the “absorption condition”). Thus, in this fMRI study we used a 2 (task: analytical vs. absorption) × 3 (video: awe vs. positive vs. neutral videos) design. Following each video, participants were required to indicate their feelings of awe, arousal, and valence (see Figure 1). In the analytical condition participants were also required to report the approximate number of perspective changes. In a previous behavioral study we found that overall participants reported stronger feelings of awe when allowed to passively watch the video compared to when counting the number of perspective changes (van Elk et al., 2016).

In line with previous studies using passive or low‐demand tasks (e.g., such as passive compared to active viewing; cf., Greicius & Menon, 2004; Shulman et al., 1997), we expected the absorption condition to be associated with increased activity in regions comprising the DMN. In contrast, the analytical compared to the absorption condition was expected to result in an increased activation of the frontoparietal attention network (FPN, including lateral brain regions such as the inferior frontal gyrus and the inferior parietal lobe; cf., Fox et al., 2006). Importantly, if watching awe compared to the control videos would fully captivate participants' attention and decrease their self‐referential thinking, we should expect that participants would be as strongly immersed in the video content of awe videos during the absorption as the analytical condition. This should be reflected in a relative decreased activation of regions comprising the DMN for awe compared to control videos presented during the absorption condition. In contrast, no differences in DMN regions are expected in the analytical condition between the different videos. Below we thus report the main effect of Task on brain activation, as well as the critical interaction‐effect—all conducted using a whole‐brain analysis with no a‐priori specification of regions of interest. In our study we also included relevant individual difference measures to determine differences in awe‐proneness (i.e., most notably the personality trait of “absorption” and “openness to experience”). At the end of the fMRI study we also conducted a posttest, during which participants were presented with the same videos, while giving more extensive ratings about their subjective experience for each video. In the posttest we also included a pictorial measure of subjective self‐perception (van Elk et al., 2016), as a proxy for the extent to which participants experienced a smaller self in association with awe videos.

## 2 METHODS

### 2.1 Participants

The experiment was conducted at the Spinoza Center for NeuroImaging at the University of Amsterdam, Amsterdam, the Netherlands. In total we enrolled 32 healthy participants in the fMRI experiment (23 females; mean age = 24.22 years, *SD* = 5.74, range = 18–41 years). Participants were recruited from the pool of participants from the University of Amsterdam, consisting mainly of students and people living in the vicinity of the University. All participants gave written informed consent and signed a screening and safety form before participation. They received 25 Euros or course credits for participation. The experiment was approved by the local ethics committee at the Psychology Department of the University of Amsterdam and the study was conducted in accordance with the guidelines from the Declaration of Helsinki.

### 2.2 Stimuli

As stimuli we used 30‐s video clips depicting different scenes and objects (see Supplementary Material Online for example stimuli). In total we used eight videos to elicit awe, eight positive videos, and eight neutral videos. Awe videos contained mainly natural scenery of impressive landscapes, such as mountains, vistas, oceans, bird flights and waterfalls. Positive videos presented various moving animals that are typically perceived to be funny or cute. Neutral videos represented more ordinary natural scenes that did not elicit awe (e.g., videos of the Dutch countryside) and moving man‐made objects (e.g., a tractor). The videos were pretested in a separate study in which 17 North American participants (eight females; mean age = 39.8 years) recruited through Amazon's Mechanical Turk, rated a larger selection of videos for arousal, valence, awe, familiarity and the feeling of “chills” on a visual analog scale ranging from 1 (“not at all”) to 100 (“very much).^1^ On the basis of the averaged ratings for each video, we selected eight videos per category such that awe videos and positive videos were matched for valence, arousal and familiarity, while they differed on feelings of awe and chills (see Table 1). As expected, both positive videos and awe videos were rated as more arousing and more positive than neutral videos.

**Table 1:** Ratings from the pretest for awe, positive, and neutral videos that were used in the main experiment and the statistical contrasts between the different categories of videos (a = awe; P = positive; N = neutral). Standard errors are between brackets. ^*^ *p* < .05; ^**^ *p* < .001

	Arousal	Valence	Familiarity	Awe	Chills
Awe videos	43.9	84.9	2.1	73.5	40.4
	(8.7)	(3.1)	(1.3)	(4.4)	(15.5)
Positive videos	45.9	84.2	2.0	35.5	11.6
	(2.1)	(4.0)	(1.7)	(8.1)	(3.1)
Neutral videos	24.6	70.4	1.6	25.4	9.1
	(3.5)	(7.6)	(1.5)	(7.8)	(3.4)
Contrasts				A > P^**^	A > P^**^
		A > N^**^	A > N^**^	A > N^**^	A > N^**^
		P > N^**^	P > N^**^	P > N^**^	P > N^*^

### 2.3 Experimental design and procedure

The experiment consisted of a single 1.5 hr experimental session for each participant. The experimental flow and procedure are presented in Figure 1. Before the start of the study participants were instructed about the experimental procedure. They then completed four practice trials outside the MRI scanner, in which we presented videos that were not included in the main experiment. Prior to the start of the experiment, participants were instructed that they would see different movies during the study and that prior to each movie they would be instructed about their task. In the *analytical condition*, participants were instructed to count the number of perspective changes by directing their attention to how often the camera changed perspective and counting the transitions between the different movie clips. In the *absorption condition*, participants were instructed to passively observe the video, including the images and sounds presented.

Following each video participants were asked to provide a rating of (a) their feelings of awe while watching the video, (b) the arousal of the video, and (c) the valence of the video on a 7‐point scale (1 = not at all, 7 = very much; 1 = negative, 7 = positive). For the awe ratings, we asked participants to what extent they felt “awe or wonder” while watching the videos. This was done because the word “awe” in Dutch (“ontzag”) is infrequent in common language use and associated with reverence and respect for authority. Typically, “wonder” refers to the more reflective aspects of spiritual and self‐transcendent experiences (Fuller, 2009). The more extensive and well‐validated post‐fMRI questionnaire showed a strong alignment with the awe ratings collected during the fMRI study. In addition, in the *analytical condition* participants were required to report the approximate number of perspective changes that they counted (i.e., 0, 1–2, 3–4, 5–6, 7–8, 9–10, 11–12, >12). While in the scanner, participants responded by using the three buttons of a response box: they could select the rating by using their index and ring finger to move a yellow circle to the left or the right side of the screen and confirmed their choice by pressing the middle button with their middle finger.

Each trial started with a fixation cross, presented for 1,000 ms, followed by a cue instructing participants about the upcoming condition (absorption or analytical). The cue was presented for a pseudo‐randomized interval of 1,000–6,500 ms in 500‐ms jittered steps. Next, a 1,000 ms fixation cross was presented followed by a 30 s video. Following the video a fixation cross appeared for 1,000 ms and depending on the condition, three (absorption condition) or four (analytical condition) ratings were subsequently presented until the subject responded to each rating. Each rating was followed by a 500 ms interval. Ratings were always presented in the same order to facilitate interpretation and responding.

Stimuli and instructions (absorption vs. analytical) were presented in a randomized order throughout the experiment. The videos were presented in two blocks of 12 videos each, separated by a 16 s break. For each participant, four videos from each stimulus category (awe, positive, or neutral) were randomly assigned to the absorption or the analytical condition. The number of times that each video was assigned to the analytical (compared to the absorption) condition is presented in Table 2. A total of 24 videos were presented according to the following design: 3 Video types (awe, positive or neutral) × 2 Instructions (absorption vs. analytical) and 4 videos per experimental condition.

**Table 2:** Number of times that each video (awe, Pos = positive videos; Ntr = neutral videos) was assigned to the analytical (compared to the absorption) condition for all participants. The proportion of videos presented in the analytical condition is presented between brackets

	Video 1	Video 2	Video 3	Video 4	Video 5	Video 6	Video 7	Video 8
Awe #	14 (0.45)	16 (0.52)	14 (0.45)	14 (0.45)	22 (0.71)	17 (0.55)	16 (0.52)	11 (0.36)
Pos #	12 (0.39)	17 (0.55)	16 (0.52)	18 (0.58)	16 (0.52)	18 (0.58)	15 (0.48)	12 (0.39)
Ntr #	17 (0.55)	17 (0.55)	14 (0.45)	15 (0.48)	13 (0.42)	13 (0.42)	15 (0.48)	20 (0.65)

Stimuli were presented using Presentation software (Neurobehavioral systems, Albany, CA). In total, fMRI data acquisition during the experimental task lasted about 40 min plus 7 min to acquire an anatomical T1 scan. In addition to the present study, participants also completed an unrelated task (on the effects of ambivalence on decision making) in the fMRI scanner and an unrelated postexperimental task after the fMRI experiment was finished. We also collected heart rate and respiration data during the fMRI scanning using the BrainProducts BrainAmp ExG MR amplifier for physiological measurements. These data were not included in the present analysis, because the main aim was to identify the neurocognitive mechanisms associated with the experience of awe.

After the fMRI experiment was finished, participants completed a survey. The individual difference measures that were included are described in more detail in the Supplementary Material Online. In the posttest we asked participants to provide more extensive ratings of each video using 9 items related to the awe experience (e.g., “To what extent did you have the feeling of transcending yourself while watching the video?”; see Table 3). We also included a pictorial scale to assess people's subjective perception of their self (see: van Elk et al., 2016). No additional measures were included in this study beyond the dependent variables and questionnaires described here.

**Table 3:** Questions to assess feelings of awe in the postexperimental test

1. To what extent did watching the video induce the experience of something beautiful?
2. To what extent did the video induce the feeling that ultimately all life is one?
3. To what extent did the video induce feelings of self‐transcendence?
4. To what extent did you experience a loss of sense of space and time during watching the video?
5. To what extent did the video induce the feeling that our life is part of a bigger whole?
6. To what extent were you impressed by watching the video?
7. To what extent did the video induce feelings of awe?
8. To what extent did you have an esthetic experience while watching the video?
9. To what extent did you have a goosebump feeling while watching the video?

We checked whether participants followed the instruction to count the number of perspective changes in the analytical condition by investigating the accuracy in the number of perspective changes detected. As participants were required to report the number of perspective changes in bins (i.e., 0, 1–2, 3–4, 5–6, 7–8, 9–10, 11–12, >12), we investigated whether there was a match between the bin identified by the participant and the bin (±1 to allow for “missed” or “ambiguous” perspective changes) that was classified for each video by the experimenter. The videos did not differ in the average number of perspective changes (awe videos: *M* = 5.5, *SD* = 2.4; positive videos: *M* = 6.4, *SD* = 4.2; control videos: *M* = 4.0, *SD* = 1.1; *t*[7] < 1.8, *p* > .12).

### 2.4 fMRI data acquisition and analysis

Imaging was performed on a 3T Philips Achieva scanner using a 32‐channel head coil. A T1‐weighted anatomical sagittal sequence image of the whole brain was acquired (repetition time [TR] = 8.2 ms; echo time [TE] = 3.8 ms; flip angle [FA] = 8°; FOV = 240*188 mm). Functional images were acquired using a T2*‐weighted gradient echo imaging sequence (EPI) to maximize the blood oxygen level‐dependent (BOLD) contrast (TR = 2,000 ms, TE = 27.63 ms; FA = 76.1°). Each volume consisted of 37 slices acquired parallel to the anterior–posterior commissure plane (ascending acquisition; voxel size = 3^3 mm; slice gap = 0.3) and for each participant a total of approximately 600 volumes were acquired (the total duration of the experiment ranged from 18.2 min to 21.7 min because of individual differences in participant's response speed).

Statistical analyses were conducted using SPM12 software (Wellcome Department of Cognitive Neurology, London, UK). Raw EPI data were preprocessed by applying slice‐time correction for differences in slice acquisition time, using the middle slice as reference slice. We performed spatial realignment to correct for effects of motion over time, and spatial normalization and smoothing with a Gaussian kernel of 5 mm FWHM (full width at half‐maximum). Anatomical normalization to MNI space was performed by coregistration of the functional images with the anatomical T1 scan.

First‐level fMRI analysis was performed for each individual subject according to the general linear model (GLM). The stimulus onset for the fMRI time‐series was fitted in one statistical model, with six regressors modeling the videos as boxcar functions convolved with the HRF, according to our 2 (Task: absorption × analytical) × 3 (Video: awe, positive, neutral) experimental design. First‐order temporal derivatives were included because of the event‐related nature of the task, as well as six additional nuisance regressors to remove residual movement artifacts. We also included the onset of the task instruction and the ratings presented between the different videos as regressors of no interest in the model.

After estimation of the first model, beta values were taken to the second level, by using a factorial design analysis to obtain a population estimate. The data were high‐pass filtered using a cutoff of 128 s to correct for baseline drifts in the signal. The coordinates of all activations are reported in MNI space and region names were identified using the Harvard‐Oxford atlas of brain anatomy (Craddock, James, Holtzheimer, Hu, & Mayberg, 2012). Contrasts of interest included the main effect of Task (i.e., absorption > analytical; analytical > absorption) and the interaction between Task and Video. We used an implicit baseline for the different contrasts and we looked at the relative difference in brain activation between the different tasks and videos (i.e., no “control condition” was included). The contrasts were thresholded at *p* < .05 using familywise error (FWE) correction for multiple comparisons at the voxel level—we assessed activity at the whole brain level and no region‐of‐interest analyses were conducted. An anatomical representation of significant clusters was obtained for visualization purposes by superimposing the structural parametric maps on a standard MNI template.

Next to the analyses reported in the present manuscript, we also conducted additional exploratory analyses to probe individual differences in the experience of awe, as well as the parametric effects of self‐reported awe on neural activity. We also conducted an additional analysis to test to what extent our effects of interest were driven by the inclusion of the different control conditions (i.e., positive and neutral videos). These analyses are described in the Supplementary Online Material.

## 3 RESULTS

### 3.1 Behavioral results

#### 3.1.1 Video ratings

**Figure 2:**
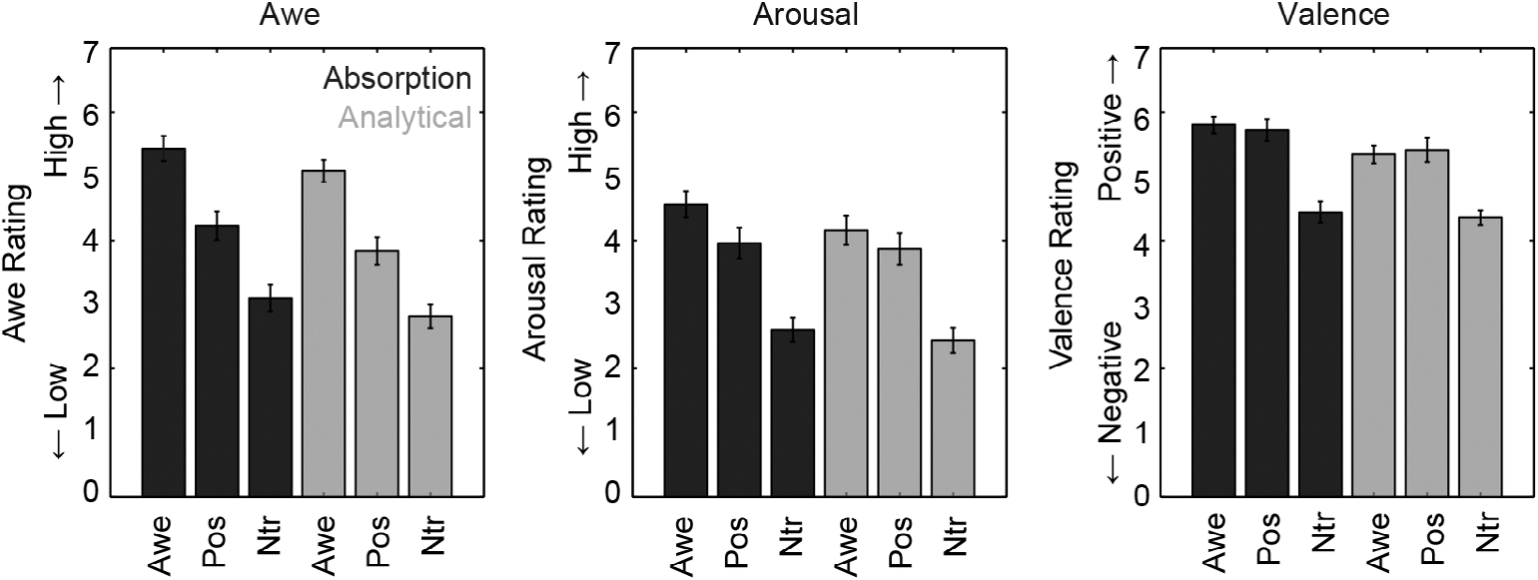
Subjective ratings for awe (left graph), arousal (middle graph), and valence (right graph) for the different videos (awe = awe videos; Pos = positive videos; Ntr = neutral videos) and the different tasks (dark bars = absorption; light bars = analytical condition). Error bars represent standard errors

The ratings of the different videos for each task (i.e., absorption vs. analytical) are presented in Figure 2. The behavioral ratings for awe, valence and arousal in response to the different videos were analyzed using a repeated measures ANOVA with Task (absorption vs. analytical) and Video (awe, positive, neutral) as within‐subjects factors.

For the *awe ratings* a main effect of Video was found, *F*(2, 62) = 60.96, *p* < .001, *η* ^*2*^ = .66, reflecting the fact that, as expected, participants experienced more awe while watching the awe videos (mean = 5.26) compared to the positive videos (mean = 4.04), *t*(31) = 5.29, *p* < .001, and compared to the neutral videos (mean = 2.96), *t*(31) = 10.85, *p* < .001. Positive videos were rated as more awe inducing than the neutral videos, *t*(31) = 6.03, *p* < .001. A main effect of Task, *F*(1, 31) = 15.16, *p* < .001, *η* ^2^ = .33, reflected higher awe ratings in the absorption condition across videos (mean = 4.26) compared to the analytical condition (mean = 3.91). The interaction between Video and Task was not significant (*F* < 1, *p* = .87).

For the *arousal ratings* a main effect of Video, *F*(2, 62) = 63.26, *p* < .001, *η* ^2^ = .67, was found, reflecting the fact that participants experienced more arousal while watching awe videos (mean = 4.36) compared to the positive videos (mean = 3.91), *t*(31) = 2.38, *p* = .024, and compared to the neutral videos (mean = 2.52), *t*(31) = 11.03, *p* < .001. In addition, the positive videos were rated as higher on arousal compared to the neutral videos, *t*(31) = 8.95, *p* < .001. Again, a significant effect of Task, *F*(1, 31) = 7.81, *p* = .009, *η* ^2^ = .21, reflected participants experiencing more arousal in the absorption condition (mean = 3.71) compared to the analytical condition (mean = 3.49). The interaction between Task and Video was not significant (*F* < 1.7, *p* = .18).

For the *valence ratings* a main effect of Video was found, *F*(2, 62) = 56.99, *p* < .001, *η* ^2^ = .65, reflecting that participants perceived awe videos as higher in valence (mean = 5.57) compared to neutral videos (mean = 4.45), *t*(31) = 12.67, *p* < .001. Positive videos were also rated as higher on valence (mean = 5.57) than neutral videos, *t*(31) = 8.61, *p* < .001. Awe and positive videos did not differ in valence ratings (*p* = .98). A main effect of Task, *F*(1, 31) = 11.82, *p* = .002, *η* ^2^ = .28, reflected that participants rated the videos as more positive in the absorption condition (mean = 5.33) compared to the analytical condition (mean = 5.04). The interaction between Task and Video was not significant (*F* < 2.19, *p* = .12).

#### 3.1.2 Postexperimental survey

The ratings from the posttest also confirmed the findings from the main study: a main effect of Video, *F*(2, 60) = 60.08, *p* < .001, *η* ^2^ = .67 reflected that participants experienced more awe (as assessed by nine different awe‐questions; see Table 2) when watching awe videos (mean = 3.36, *SE* = .15), compared to positive videos (mean = 2.18, *SE* = .16), *t*(30) = 7.14, *p* < .001, and compared to neutral videos (mean = 1.89, *SE* = .11), *t*(30) = 11.32, *p* < .001. With respect to the pictorial self representation measure (which was conducted after the fMRI study had been completed), we found a main effect of Video, *F*(2, 60) = 26.21, *p* < .001, *η* ^2^ = .47, reflecting that participants perceived their self to be smaller when watching awe videos (mean = 4.01, *SD* = 1.92) compared to positive videos (mean = 5.35, *SD* = 1.31), *t*(30) = −5.32, *p* < .001, and compared to control videos (mean = 5.12, *SD* = 1.37) *t*(30) = −5.17, *p* < .001.

#### 3.1.3 Characteristics of videos

When looking specifically at participants' responses to the different questions, we found that the awe videos were rated specifically high on Question 1 (“To what extent did watching the video induce the experience of something beautiful?”), Question 6 (“To what extent were you impressed by watching the video?”) and Question 8 (“To what extent did you have an aesthetic experience while watching the video?”). This reflects that participants primarily had an aesthetic experience while watching the awe videos: they felt impressed by something beautiful and profound.

Furthermore, when looking at the averaged awe ratings for each of the different awe videos, we found that there was only limited variability in the amount of awe reported for each video (range = 3.00–3.38). The strongest awe‐inducing videos consisted of footage from BBC Earthflight and BBC Planet Earth.

#### 3.1.4 Perspective taking task

Participants made quite a lot of errors when counting the number of perspective changes in the analytical condition (mean accuracy = 65.6%; *SE* = 3.4%).^2^ For the accuracy of the counting task, a main effect of Video was found, *F*(2,62) = 4.66, *p* = .013, *η* ^2^ = .13. Participants made more errors when counting the number of perspective changes for awe videos (mean = 56.3%, *SE* = 5.0%), compared to positive videos (mean = 73.4%, *SE* = 3.7%), *t*(31) = −2.82, *p* = .008, and compared to neutral videos (mean = 67.2%, *SE* = 5.0%), *t*(31) = −2.08, *p* = .046.

### 3.2 fMRI results

#### 3.2.1 Main effects of task

**Figure 3:**
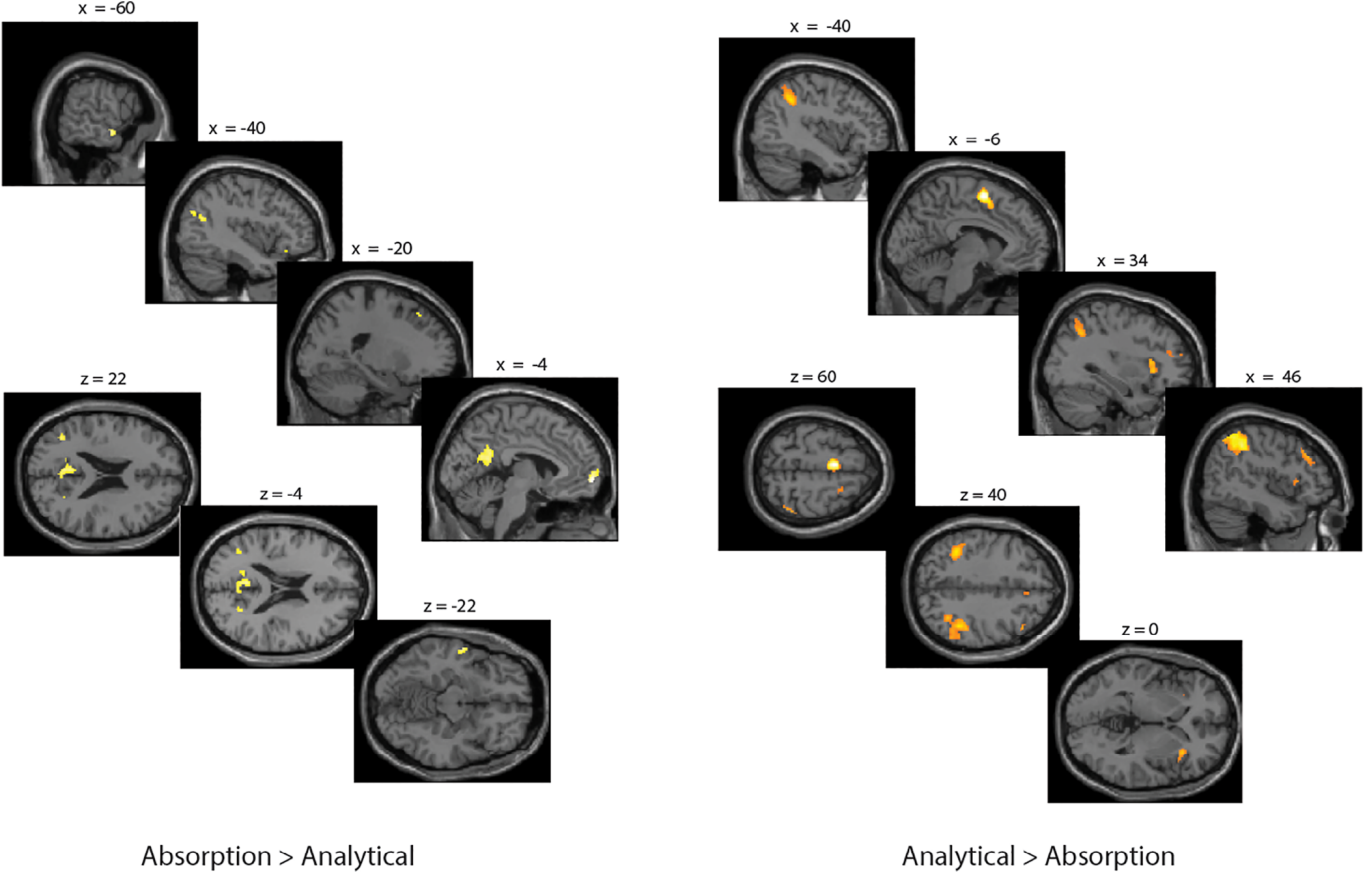
Activation maps representing areas that showed a stronger activation for the absorption compared to the analytical condition (left side) and for the analytical compared to the absorption condition (right side). Results are thresholded at *p* < .05 FWE‐corrected at the whole‐brain level. Error bars represent standard errors [Color figure can be viewed at http://wileyonlinelibrary.com]

Using a whole‐brain analysis, comparing trials in which participants were passively immersed in the video compared to trials in which they counted the number of perspective changes (absorption > analytical) revealed increased activation in the frontal pole extending into the superior frontal gyrus, the bilateral middle temporal gyri, the posterior cingulate cortex, the lateral occipital cortex and the temporal pole (see Table 4 and Figure 3 left side).

**Table 4:** Activation of brain regions for the different contrasts as a function of task instruction. The upper part of the table reflects brain regions showing stronger activity for the absorption compared to the analytical condition; the lower part reflects the reverse contrast (analytical > absorption)

Absorption > analytical						
Regions	Hemi	*x*	*y*	*z*	*t*	Voxels
Frontal pole	Left	−4	62	−6	7.30	144
Middle temporal gyrus	Right	60	−2	−16	6.89	27
Middle temporal gyrus	Left	−60	−2	−12	6.46	52
Posterior cingulate cortex	Left	−2	−52	28	6.29	274
Superior frontal gyrus	Left	−20	30	52	5.87	22
Frontal pole	Left	−8	46	42	5.82	14
Angular gyrus	Left	−40	−62	22	5.82	32
Precuneus	Right	26	−60	20	5.68	12
Temporal pole	Left	−46	10	−30	5.62	13
Angular gyrus	Left	−40	−72	28	5.33	15

Analytical > absorption						
Regions	Hemi	*x*	*y*	*z*	*t*	Voxels
Supplementary motor cortex	Left	−6	2	60	11.65	440
Supramarginal gyrus	Right	46	−42	46	9.35	1,134
Middle frontal gyrus	Right	42	32	34	7.47	298
Supramarginal gyrus	Left	−40	−46	40	7.39	510
Insula	Right	34	22	0	6.64	188
Cerebellum	Left	−32	−66	−32	6.42	102
Superior frontal gyrus	Right	24	8	62	5.78	27
Insula	Left	−28	22	4	5.62	16

The reverse contrast (analytical > absorption) showed a stronger activation of the supplementary motor cortex, the supramarginal gyrus, the middle frontal and superior frontal gyrus and the insula (see Table 4 and Figure 3 right side).

#### 3.2.2 Interaction effects between Task & Video: absorption > analytical

**Figure 4:**
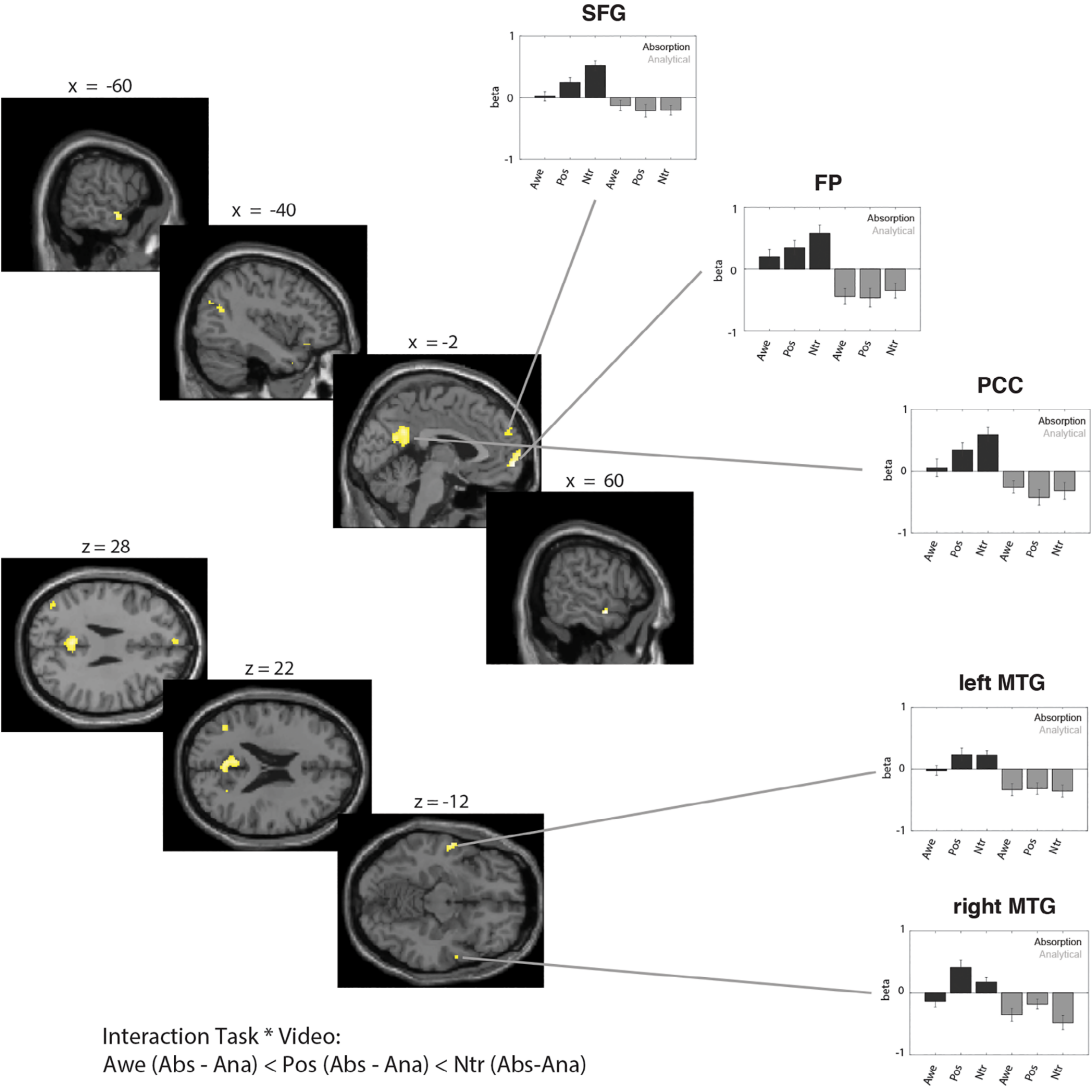
Interaction effect between task and video: absorption > analytical. Activation maps (left side) and beta‐estimates (right side) for areas that showed a differential activation as a function of both task (absorption vs. analytical) and video (awe, positive, or neutral). Specifically, we probed for the contrast whereby the difference between the absorption and the analytical condition was smaller for the awe compared to the positive and for the positive compared to the neutral condition. Results are thresholded at *p* < .05 FWE‐corrected at the whole‐brain level. Error bars represent standard errors. Although all significant clusters of course show an increase in the difference between the absorption and analytical condition going from awe to positive to neutral conditions, the specific pattern of response amplitude differed substantially between ROIs, as can be seen in the individual beta‐estimates extracted from the different clusters [Color figure can be viewed at http://wileyonlinelibrary.com]

Using a whole‐brain analysis, we examined the interaction between Task and Video to investigate whether the activity during the absorption compared to the analytical task differed between videos. We specifically probed for the contrast whereby the difference between the absorption and the analytical condition was smaller for the awe compared to the positive and for the positive compared to the neutral condition; thus the critical contrast was specified as follows: awe (absorption – analytical) < positive (absorption – analytical) < neutral (absorption – analytical). We found that the activation of a cluster of brain regions in association with the absorption instruction was strongest in the neutral video condition, as compared to the positive video condition, and smallest in the awe video condition (see Table 5 and Figure 4). The brain areas showing a differential activation as a function of task and video included the frontal pole, extending to the superior frontal gyrus, the posterior cingulate cortex, the bilateral middle temporal gyri, the left temporal pole and the angular gyrus. Although all significant clusters of course show an increase in the difference between the absorption and analytical condition going from awe to positive to neutral conditions, the specific pattern of response amplitude differed substantially between ROIs, as can be seen in the individual beta‐estimates extracted from the different clusters.

**Table 5:** Interaction‐effect absorption > analytical: brain regions showing a differential activation as a function of task (absorption vs. analytical) and video (awe, positive, and neutral)

Interaction task * video: absorption > analytical							
Regions	Hemi	*x*	*y*	*z*	*t*	Voxels	Within DMN
Middle temporal gyrus	Right	60	−2	−16	7.16	39	Adjacent (nearest cluster *x* = 60 *y* = −4 *z* = −20)
Frontal pole	Left	−4	62	−6	7.13	146	Yes
Posterior cingulate cortex	Left	−2	−52	28	6.64	362	Yes
Middle temporal gyrus	Left	−60	−6	−12	60	60	Adjacent (nearest cluster *x* = −60 *y* = −6 *z* = −14)
Frontal pole	Left	−8	46	42	6.18	76	Adjacent (nearest cluster *x* = −4 *y* = 50 *z* = 42)
Temporal pole	Left	−46	10	−30	5.91	24	Yes
Angular gyrus	Left	−40	−62	22	5.71	27	Adjacent (nearest cluster *x* = −44 *y* = −64 *z* = 22)
Angular gyrus	Left	−42	−72	28	5.48	10	Yes
Superior frontal gyrus	Left	−4	54	28	5.47	23	Yes

To probe whether these regions could be considered part of the default mode network, we compared the loci of activation with the coordinates provided by Neurosynth, which integrates findings from 1,335 term‐based meta‐analyses of neuroimaging articles (http://neurosynth.org; we used the key term “default” which resulted in the inclusion of 907 studies and a total of 31,297 activations). We found that the key regions showing a differential activation as a function of task and video, fell well within or were adjacent to the DMN—as identified by the coordinates reported in the literature (see Table 5).

We conducted a post‐hoc test to directly determine whether the effects of Video on DMN activity differed between the absorption and the analytical condition. To this end we first exported the beta‐values for the key regions that were found activated in the interaction‐contrast (i.e., the FP, PCC and the SFG). We subjected the beta‐values to two repeated measures ANOVA with the factors brain region (three levels) and video (awe, pos, ntr) separately for the absorption and the analytical condition. We found a main effect of Video in the Absorption condition, *F*(2, 62) = 5.04, *p* = .009, *η* ^2^ = .14, while this effect was not significant in the Analytical condition, *F*(2, 62) = .41, *p* = .66. Post‐hoc *t*‐tests showed that within the absorption condition, awe videos differed from positive videos, *t*(31) = −2.25, *p* = .031 and from neutral videos as well, *t*(31) = −3.08, *p* = .004. Positive videos did not differ from neutral videos, *t*(31) = −1.83, *p* = .078.

In an additional analysis we also tested to what extent the interaction‐effect between Task and Video was driven by the inclusion of specific control conditions (i.e., positive or neutral videos). This analysis is reported in the Supplementary Material Online.

#### 3.2.3 Interaction effects between Task & Video: analytical vs. absorption

**Figure 5:**
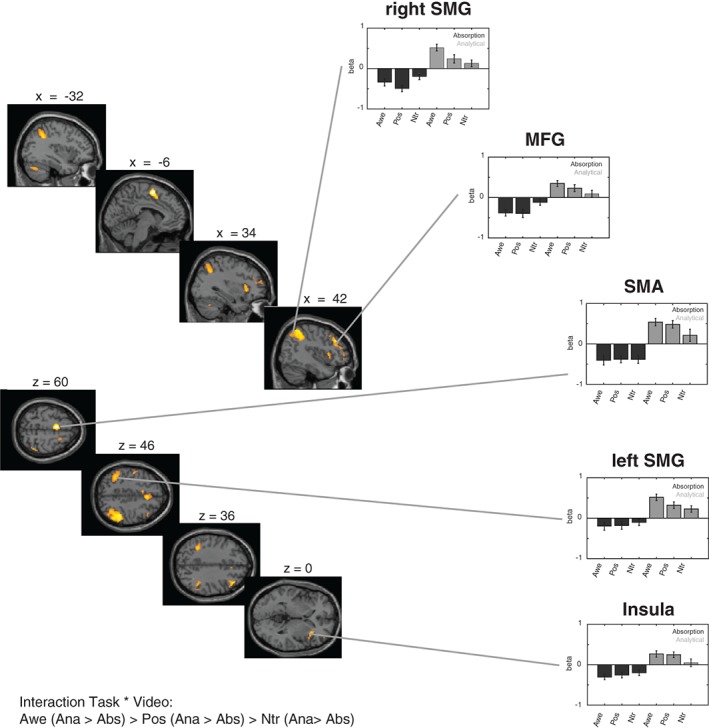
Interaction effect between task and video: analytical > absorption. Activation maps (left side) and beta‐estimates (right side) for areas that showed a differential activation as a function of both task (absorption vs. analytical) and video (awe, positive, or neutral). The figure presents the contrast whereby the difference between the analytical and the absorption condition was larger for the awe compared to the positive and for the positive compared to the neutral condition. Results are thresholded at *p* < .05 FWE‐corrected at the whole‐brain level. Error bars represent standard errors [Color figure can be viewed at http://wileyonlinelibrary.com]

In a second analysis we probed for the contrast whereby the difference between the analytical and the absorption condition was larger for the awe compared to the positive and for the positive compared to the neutral condition; thus the contrast was specified as follows: awe (analytical – absorption) > positive (analytical – absorption) > neutral (analytical – absorption). This interaction‐effect showed a differential activation as a function of task and video in core regions in the supplementary motor cortex (SMA), the supramarginal gyrus (SMG), the middle frontal gyrus (MFG), the insula and the precentral gyrus (see Table 6). As can be seen in Figure 5 these regions were most strongly activated in the analytical condition when watching awe videos, compared to positive videos and neutral videos.

**Table 6:** Interaction effect analytical > absorption: Brain regions showing a differential activation as a function of task (absorption vs. analytical) and video (awe, positive and neutral)

Interaction task * video: analytical > absorption						
Regions	Hemi	*x*	*y*	*z*	*t*	Voxels
Supplementary motor cortex	Left	−6	2	60	11.70	562
Supramarginal gyrus	Right	46	−42	46	9.46	1,308
Middle frontal gyrus	Right	42	32	36	7.99	432
Supramarginal gyrus	Left	−36	−48	38	7.62	571
Cerebellum	Left	−32	−70	−30	7.11	171
Insula	Right	34	22	0	6.97	24
Superior frontal gyrus	Right	24	8	62	5.72	40
Precentral gyrus	Left	−50	−8	46	5.64	24
Cerebellum	Right	38	−56	−32	5.46	25
Insula	Left	−30	22	4	5.43	25
Middle frontal gyrus	Left	−42	30	32	5.15	12

To probe whether these regions could be considered part of the frontoparietal attention network (FPN; cf., Naghavi & Nyberg, 2005), we compared the loci of activation with the coordinates provided by Neurosynth: we used the key term “frontoparietal” which resulted in the inclusion of 360 studies and a total of 13,467 activations). We found that the key regions showing a differential activation as a function of task and video, fell well within the FPN—as identified by the coordinates reported in the literature—except for the activation in the SMA, which in our study was likely related to overt counting in the analytical condition.

## 4 DISCUSSION

In this fMRI study we tested the hypothesis that the experience of awe would be associated with reduced self‐referential processing, which would be accompanied by a reduced activation of brain areas that are considered part of the default mode network (DMN) when watching awe videos compared to control videos. We orthogonally manipulated the level of immersion in external stimuli and the type of stimuli that were presented, using awe, positive, and neutral videos. The subjective ratings showed that our manipulation was successful: participants experienced stronger feelings of awe when watching awe compared to control videos, and when watching the videos with an absorptive compared to an analytical mindset. Participants also indicated that they subjectively perceived a smaller self when watching awe compared to control videos.

At a neural level, by using a whole‐brain analysis we found that the frontal pole (FP), the posterior cingulate cortex (PCC) and the angular gyrus (AG) were less strongly activated when participants were watching awe videos compared to control videos in the absorption condition (i.e., when participants were passively watching the videos without an explicit task). In contrast, no differential activation of these brain areas was observed when participants were watching the different videos in the analytical condition (i.e., when participants were actively counting the number of perspective changes). Based on a comparative analysis using Neurosynth (http://neurosynth.org), we found that five peak coordinates of our critical interaction‐effect between task and video, fell within previously reported coordinates of the DMN, while four peak coordinates were adjacent to the DMN (see Table 5). We did not include an independent localizer in our study to probe the overlap between DMN regions and the interaction‐effect between task and video. Accordingly, we warrant caution in interpreting our findings as directly reflecting reduced DMN activity for awe videos. Still, the reduced activity of the FP the PCC and the AG in the absorption condition for awe‐videos (i.e., awe < positive < neutral) fits well with the suggestion that the increase in DMN regions that is usually found in passive task conditions (Naghavi & Nyberg, 2005), is strongly reduced in the case of awe. Below we discuss the behavioral and neuroimaging findings in more detail and reflect on the theoretical implications of this result for our understanding of the experience of awe.

The subjective awe ratings indicate that our experimental manipulation was successful: participants experienced more awe when presented with awe inducing compared to positive and neutral videos and when they were allowed to get passively immersed in the videos compared to when counting the number of perspective changes. Previous studies have reported similar effects of task instruction and type of video on subjectively experienced awe (Greicius & Menon, 2004; Shulman et al., 1997), indicating that this method provides a reliable way to experimentally manipulate feelings of awe in the lab. We also note that the absolute awe ratings were quite high (5.5 on a 7‐point scale), indicating that despite the artificial environment and the scanner noise, participants still experienced quite profound feelings of awe. Using self‐report measures we also found that while watching awe videos, participants perceived their self to be smaller. This finding provides complementary support for the suggestion that feelings of awe are characterized by a reduced focus on the self, which is reflected in a relative decreased activity of the DMN.

At a neural level, we found an increased activation in brain regions such as the FP, the PCC and the AG when participants were passively absorbed in the video. Based on the available data in Neurosynth (http://www.neurosynth.org), we were able to establish that our clusters of activation fell within or were adjacent to the DMN, as it has been identified and localized in previous studies and meta‐analyses. In contrast, the instruction to count the number of perspective changes activated regions that have been associated with the frontoparietal attention network (FPN) (Naghavi & Nyberg, 2005), such as the SMG, the MFG, and the insula.

In the existing literature different possible functions of the DMN and the FPN have been proposed (M. D. Fox et al., 2005). It has been suggested that DMN activity supports spontaneous cognition (e.g., self‐referential processing and mind‐wandering), while the FPN is primarily involved in goal‐directed (i.e., task‐related) cognition (Jack et al., 2013). In addition, whereas the DMN has been associated with internally directed attention (i.e., focusing on one's own thoughts and imagery), the FPN has been related to externally directed attention (Raichle, 2015). Our results converge with these suggestions. We found that brain regions of the DMN were more strongly activated when participants were passively watching a video and that brain regions of the FPN were more activated during the analytical condition.

The stronger DMN activity that we observed for the absorption compared to the analytical condition could be related to a stronger engagement in spontaneous cognition (i.e., mind‐wandering and self‐referential processing). Importantly, when participants were watching the videos in the absorption condition, the activation of core areas of the DMN was strongest for neutral videos and weakest for awe videos. We speculate that watching neutral videos in the absorption condition was probably less engaging than watching positive or awe videos, and accordingly participants may have engaged in mind‐wandering and self‐referential processing to a greater extent in the neutral condition. In contrast, it may have been easier for participants to get absorbed in the awe videos, resulting in reduced self‐referential processing. In this study we did not include a direct measure of mind‐wandering (e.g., by using the experience‐probing technique), nor did we measure ease (or difficulty) of doing the experimental tasks. Our behavioral findings in the analytical condition, however, indicate that participants made most errors in counting while watching awe videos. Furthermore, we found that activity in the FPN differed as a function of video: key regions of the FPN, such as the SMG, the MFG, and the insula were most strongly activated in the analytical condition when participants were watching awe compared to positive and neutral videos. These findings—next to the subjective ratings from the posttest—provide further support for the notion that awe‐videos strongly appealed to participants and captured their attention, even to the extent that participants required more attentional control when counting the number of perspective changes in awe‐videos than in positive videos and neutral videos. We consider a similar process of attentional capture and absorption to be responsible for the reduction in self‐referential processing as reflected in the DMN and the pictorial self representation measurements.

Crucially, the key regions of the DMN including the FP, the PCC, and the AG were not differentially activated as a function of video in the analytical condition. This finding rules out the potential confound that the effects on DMN activity in the absorption condition were driven by low‐level visual and auditory differences between the videos. Still, it could be that participants found it relatively easier to get immersed in the awe videos compared to the positive and the neutral videos, and that ease of immersion in turn determined activity in the DMN. A key feature of many awe‐eliciting stimuli is that they concern events that support immersion (be it a concert, an artwork or a beautiful nature scenery). Our findings indeed suggest that—irrespective of the task instruction—the DMN was not as strongly activated while watching awe‐videos, compared to watching positive and neutral videos (see Figure 5). Thus, a key feature of awe experiences may be the potential to get passively and effortlessly immersed in external stimuli (Buckner et al., 2008). Behavioral studies may further confirm this hypothesis, by paying more detailed attention to the subjective phenomenological reports of the awe experience by participants (Lifshitz, van Elk, & Luhrmann, resubmitted).

## 5 CONCLUSIONS

We found that the increase in key regions of the DMN during the absorption condition differed with the type of video presented: watching awe‐videos resulted in less activity of these regions compared to positive and neutral videos. These findings provide neurocognitive support for the hypothesis that the experience of awe is associated with reduced self‐referential processing. More specifically, our findings suggest that absorption in awe may be accompanied by a reduction in mind‐wandering and spontaneous self‐reflective thought that is comparable to active engagement in attentional or analytical tasks. In contrast, key regions of the FPN were more strongly activated when watching awe compared to control videos in the analytical condition. This finding underlines the captivating, immersive and attention‐grabbing nature of awe stimuli that is considered to be responsible for reductions in self‐reflective thought. In sum, our study confirms and elucidates existing theoretical accounts that consider the relationship between awe and the self and provides new insights in the neurocognitive mechanisms underlying the experience of awe.

6

## Supporting information


**Appendix S1:** Supplementary Material Online

**Video S1**

**Video S2**

**Video S3**

## Data Availability

The data that support the findings of this study are available from the corresponding author upon reasonable request.

## Footnotes

1 We note that the MT sample may be different from the Amsterdam cohort that was tested in the main study, which could explain the slightly different ratings that we obtained for the fMRI experiment.

2 Note that for some videos it was unclear whether some transitions qualified as a “perspective change”; accordingly we classified participants' responses as correct if their classification was within ±1 bin with respect to the bin identified by the researchers.
