# Supplementary material for: The neural correlates of the awe experience: Reduced default mode network activity during feelings of awe
Source: Hum Brain Mapp. 2019 May 7;40(12):3561–74. doi: 10.1002/hbm.24616 (PMC6766853; doi:10.1002/hbm.24616)
Supplement: Supplementary file 1 — Appendix S1: Supplementary Material Online [file HBM-40-3561-s001.doc]

**Supplementary Material Online**

*Relation of Awe with Individual Difference Measures*

The Tellegen absorption scale (Tellegen & Atkinson, 1974) measures the tendency to get absorbed in one’s internal imagery or external sensory events (example items include for instance: ‘When I listen to music I can get so caught up in it that I don't notice anything else.’ And: ‘I can be deeply moved by a sunset.’). The Tellegen scale consists of 34 items and is answered on a 5-point scale ranging from 1 (not at all) to 5 (very much; Cronbach’s α = .94). Based on previous findings (van Elk, Karinen, Specker, Stamkou, & Baas, 2016) we hypothesized that the Tellegen Absorption scale could act as a potential moderator of our experimental manipulation. We included the Tellegen Absorption scale as a covariate in the Repeated Measures analysis while using the behavioral awe-ratings as dependent variable. A significant interaction was found between Task and Absorption, *F*(1, 30) = 6.14, *p* = .019, η^2^ = .17, reflecting that participants scoring high on absorption showed a more pronounced effect of Task (i.e., mean awe rating absorption condition = 4.45, SE = .21 vs. analytical = 3.96, SE = .21) on the awe-ratings compared to participants scoring low on absorption (i.e., mean awe rating absorption = 4.04, SE = .22 vs. analytical = 3.83, SE = .22). In addition, a main effect of Absorption was found, *F*(1, 30) = 5.48, *p* = .026, η^2^ = .16, indicating that people scoring high on absorption reported stronger feelings of awe – a finding that is also consistent with previous studies (van Elk et al., 2016).

In addition to the absorption the post-experimental survey included the spiritual transcendence scale (Piedmont, 1999), the dispositional positive emotions scale (Shiota, Keltner, & John, 2006) and the openness to experience scale (McCrae, 1987) for exploratory purposes. When including the other personality scales as covariate in the analysis we found a main effect of self-transcendence, *F*(1, 28) = 8.86, *p* = .006, and a main effect of dispositional positive emotions, *F*(1, 30) = 5.71, *p* = .023, indicating that participants scoring high on these scales also had more profound feelings of awe.

*Neural Correlates of Subjective Awe Experiences*

To investigate which brain areas were modulated as a function of subjectively experienced awe we included the subjective awe rating of individual videos as a parametric regressor in the design matrix for each participant in an additional analysis. When using a conservative threshold (*p* < .05 FWE-corrected at the voxel level), no significant main effects of subjective awe ratings on brain activity were observed. At a more lenient statistical threshold we observed a cluster in the left lateral occipital cortex (x = -48, y = -74, z = 14, k_e_ = 319, t = 4.01) that showed an increased activation with higher ratings of awe. This effect could be driven however, by the specific visual content of the videos that triggered stronger feelings of awe (e.g., vast landscapes, sceneries etc.).

*fMRI Results controlling for Video*

In our main analysis reported in the paper we always included all three video conditions in the statistical test. To determine to what extent our critical effects of interest were driven by the inclusion of the neutral condition in the interaction-contrast, we also conducted an additional analysis. We tested for the interaction between Task and Video based on only the Awe and the Positive videos: Awe (Absorption – Analytical) < Positive (Absorption – Analytical). This analysis yielded a near-identical pattern of results (albeit with smaller cluster sizes; see Supplementary Table 1 below) as that of the critical interaction that we reported in the paper. When we tested the interaction using only awe and neutral videos, a similar pattern of results emerges. This additional analysis suggests that our analysis and effects of interest are robust to the specific control conditions that were included.

***Supplementary Table 1:*** *Brain regions showing a differential activation as a function of task (absorption vs. analytical) and video (awe and positive videos).*

| **Regions** | **Hemi** | **X** | **y** | **z** | **T** | **voxels** |
| --- | --- | --- | --- | --- | --- | --- |
| Frontal Pole | Left | -2 | 62 | -6 | 6.15 | 44 |
| Posterior Cingulate Gyrus | Left | -2 | -52 | 28 | 5.40 | 11 |
| Supramarginal Gyrus | Left | -60 | -32 | 32 | 5.24 | 10 |
| Middle Temporal Gyrus | Right | 60 | -2 | -16 | 5.52 | 7 |
| Middle Temporal Gyrus | Left | -60 | -6 | -12 | 5.41 | 6 |
| Precuneus | Left | -6 | -56 | 20 | 5.03 | 6 |
| Superior Frontal Gyrus | Left | -20 | 30 | 52 | 5.10 | 1 |
| Angular Gyrus | Left | -38 | -64 | 24 | 5.03 | 1 |
| Precuneus | Left | -6 | -64 | 24 | 4.87 | 1 |

**References**

McCrae, R. R. (1987). Creativity, divergent thinking, and openness to experience. *Journal of Personality and Social Psychology, 52*(6), 1258-1265.

Piedmont, R. L. (1999). Does spirituality represent the sixth factor of personality? Spiritual transcendence and the five‐factor model. *Journal of Personality, 67*(6), 985-1013.

Shiota, M. N., Keltner, D., & John, O. P. (2006). Positive emotion dispositions differentially associated with Big Five personality and attachment style. *The Journal of Positive Psychology, 1*(2), 61-71.

Tellegen, A., & Atkinson, G. (1974). Openness to absorbing and self-altering experiences (" absorption"), a trait related to hypnotic susceptibility. *J Abnorm Psychol, 83*(3), 268-.

van Elk, M., Karinen, A., Specker, E., Stamkou, E., & Baas, M. (2016). ‘Standing in Awe’: The Effects of Awe on Body Perception and the Relation with Absorption. *Collabra, 2*(1).
